# Evaluation of the effects of the body on athletes’ emotions and motivational behaviors from the perspective of big data public health

**DOI:** 10.3389/fpsyg.2025.1640081

**Published:** 2025-08-14

**Authors:** Qiang Zhang, Diandong Lian, Yiqiao Zhang

**Affiliations:** ^1^College of Physical Education, Suzhou University, Suzhou, Anhui, China; ^2^Department of Physical Education, Tarim University, Alar, Xinjiang, China; ^3^College of Physical Education, Hubei University of Arts and Sciences, Xiangyang, Hubei, China

**Keywords:** public health perspective, athlete emotion, particle swarm optimization, K-nearest neighbor, support vector machine

## Abstract

**Objective:**

An analysis was conducted on the impact of the body on athletes’ emotions and motivation from the perspective of Public Health (PH).

**Methods:**

PSO-KNN (Particle Swarm Optimization-K-Nearest Neighbor) algorithm and PSO-SVM algorithm (Particle Swarm Optimization-Support Vector Machine) were obtained by combining Particle Swarm Optimization (PSO), K-Nearest Neighbor (KNN), and Support Vector Machine (SVM), and then the recognition rates of the two algorithms were compared.

**Results:**

When comparing the PSO-KNN algorithm and PSO-SVM algorithm on baseline removed and baseline not removed, the average recognition rates of PSO-KNN algorithm and PSO-SVM algorithm under emotional state were 56.66 and 54.75%, respectively. The average recognition rates of PSO-KNN algorithm and PSO-SVM algorithm with baseline removal under tension were 53.16 and 50.58%, respectively.

**Conclusion:**

The algorithm that removes the baseline is better than the algorithm that does not remove the baseline, and the PSO-KNN algorithm is better than the PSO-SVM algorithm.

## 1 Introduction

In the current society, there is increasing attention to the psychological and emotional aspects of athletes. Athletes also pay great attention to their emotional issues during regular training, and analyze whether the quality of their emotions would affect their performance and whether it would affect their emotions when their physical condition is not good (Kopp et al., 2021; Hut et al., 2021).

From the perspective of PH, Alvidrez J analyzed the research framework of the National Institute for Health and Health Differences among Ethnic Minorities, and believed that understanding and addressing minority health and health differences, as well as promoting health equity related health, are factors determining the study of health disparities. Regarding how to use this framework to evaluate the health and health disparities of ethnic minorities, as well as future priorities, he conducted an analysis of research projects and identified gaps and opportunities for future research on minority health and health disparities to understand and address all complex health disparities and promote the need for health equity. Using a multidimensional research perspective, the research framework of the National Institute of Minority Health and Health Differences is a tool for conceptualizing and describing the promotion of minority health. These decisions reflect the underlying causes of health outcomes, as well as intervention goals to improve minority health or reduce disparities. He also believed that the framework is an ongoing work and may be modified based on changes in research concepts or terminology, as well as feedback from off campus communities and other stakeholders (Alvidrez et al., 2019). In the global response to the COVID-19 (Corona Virus Disease 2019) pandemic, Bektas A believed that even if the pandemic is ultimately controlled, the short-term and long-term sequelae after recovery from these diseases indicate that these complex symptoms lead to an accelerated clinical systemic inflammatory state. Usually, an exacerbation of inflammation can be seen in the elderly, leading to an increase and deterioration of age-related diseases, and even symptoms of inflammation can be seen in young people (Bektas et al., 2020). From the perspective of PH, people should pay more attention to their physical health. When ordinary people fall ill, their emotions also become low. Due to the low emotions, the health situation becomes more severe. Therefore, in the current society, more attention should be paid to PH.

In team sports, athletes must manage individual interests and group goals, emphasizing the complexity between personal identity and social identity. Campo M studied the impact of identity mechanisms on the evaluation process and analyzed whether the level of self-abstraction would lead to group emotions and impact performance. During the analysis process, Campo M adopted an experimental design to manipulate the level of self-abstraction by inducing self-oriented and team-oriented goals. Thirty outstanding male rugby players were simulated and a linear mixed effects model was obtained to show. High levels of self-abstraction lead to more positive and less negative individual emotions and perceived team reference emotions, reducing the correlation between team reference emotions and individual reference emotions. Campo M believed that after controlling for the potential impact of self-abstraction level, only positive teams refer to emotions as influencing performance (Campo et al., 2019). When studying the correlation between time perspective activities and individual organization, Cheban Y analyzed 27 high-level rowers and identified two experimental groups using cluster analysis. Through two sets of experimental analysis, it was found that the level of physical fitness includes responsibility, courage, self-control, and emotional balance in the development of personal qualities (Cheban et al., 2020). In this regard, positive emotions and pleasurable experiences are experienced with the same frequency and much more frequently than negative ones.

The article would also analyze athletes’ emotions and motivations from the perspective of PH by analyzing the PSO algorithm, KNN algorithm, and SVM algorithm, and combining the algorithms to get a better algorithm.

## 2 Introduction to the relationship between the body and athlete emotions from the perspective of PH

### 2.1 PH perspective

PH is a controversial concept, generally defined as the technology that is achieved through the joint efforts of society to prevent diseases, extend life expectancy, and ensure health (Wang et al., 2021). It is a combination of science, practical technology, and faith that guides the maintenance and improvement of PH (Olowe et al., 2024). PH is an organization that focuses on major health issues that affect every society, with a focus on local, ethnic, national, and international factors. It contains rich connotations. Any issues related to PH can be understood as PH issues (Guloksuz and van Os, 2018). Compared to ordinary hygiene concepts, PH has four main characteristics.

One is to value the “public” or the health of the general public, rather than individual health. Taking hypertension patients as an example, doctors often ask the question: “How did this patient get sick at this time?” However, from the perspective of PH, doctors have another question: why does this group of people have hypertension? Secondly, attention should be paid to prevention. Its fundamental principle is to prevent diseases as a whole, rather than individualized treatment and recovery. Thirdly, it covers a wide range and involves all issues in the field of PH. The fourth is a social product, and its promotion is a group activity that requires the help of social forces to achieve its goals.

### 2.2 Emotional impact of physical exercise on people

Emotion is a human mindset, and if it can be adjusted well, it would have a positive effect on people’s work, study, and life. Participating in a certain intensity of physical exercise can help college students maintain a good mood, alleviate their depression and anxiety, and have a positive impact on their mental health (Chekroud et al., 2018).

1. The promoting effect of sports on positive emotions.

There is a dialectical and unified relationship between emotions and health. A good mood can improve the quality of the body, while a good physique can promote the development of good emotions (Shen, 2021). Athletes are energetic because they have a healthy body, and they are full of energy and full of fighting spirit, which is due to a positive emotion they generate in sports.

2. The positive impact of sports on psychological activities.

In the process of sports, due to the movement of the body and changes in the external environment, the sensory organs of the body are stimulated, thereby triggering a subjective sense of consciousness in the brain (Wu, 2025). Over a period of time, a certain body receptor would repeatedly experience certain bodily movements and external stimuli, thus generating a relatively stable psychological characteristic in terms of willpower. In social practice activities, when subjected to specific stimuli, it would be reproduced again. This psychological activity reflects the internal connection between different situational stimuli, causing people to transfer the psychological characteristics triggered by sports stimuli to the performance of social stimuli (Caputo and Reichert, 2020). In 1979, a psychologist from another country used the Kotma Personality Factor Test to test college students who participated in and did not participate in sports activities. The results show that there is a significant difference in willpower between the two individuals.

The negative impact of sports on college students’ emotions:

1. The impact of students’ physical fitness on their emotions.

According to a survey, 61.4% of college students say that negative emotions only arise when their physical fitness is inferior to their peers (Wang et al., 2022). At the same time, it has been found in teaching classrooms that as long as students master the technology proficiently, it would bring good chain effects to learning. In contrast, 45–65% of students who are slow and ineffective in mastering technical movements would feel depressed and easily bored, and in some cases, they would even just practice passively to meet the teacher’s requirements and take a lazy break without paying attention.

2. The impact of teacher teaching on students’ emotions.

The teaching level of teachers is relatively low, and they are unable to accurately grasp the key points, difficulties, and teaching sequence of the teaching content. The lack of innovative practice methods, monotonous and poor explanation language, and unreasonable arrangement of exercise loads can all make students feel bored in the teaching class, leading to changes in their mood (Fletcher et al., 2018). Therefore, they are likely to develop in the direction of negative emotions.

In the process of comprehensively carrying out physical education, the authority and exemplary role of teachers also have a significant impact on students’ thoughts and psychology. Only a teacher with prestige and democracy can win the respect and love of students. In classroom teaching, the emotional state of teachers can also affect the emotional state of students (Frenzel et al., 2021).

3. The main factors and changing characteristics that affect students’ negative emotions in the process of physical education teaching.

In terms of technology, there are many types and contents of sports comprehensive quality techniques, including many different combinations of movements, different difficulties, and different contents, which can make students have different moods (Simonton et al., 2021). In communication with students from various majors, it is found that their attitudes are often positive, with 30–40% of students feeling self-satisfied. If the movements learned are difficult, students’ mastery of these movements is slow or difficult to complete. 10–15% of students avoid or perfunctorily approach things for various reasons and do not impose strict requirements on themselves.

Tactics: In the process of teaching comprehensive physical education, 20–40% of students often make technical and cooperative mistakes during comprehensive exercises or team teaching competitions, which can easily lead to negative emotions such as resentment and anger. Although some students do not use language, facial expressions, or physical movements to express their emotional changes in class, according to a survey, 50–60% of students still experience negative emotions. Because in the teaching of comprehensive physical education, the characteristics of high teamwork requirements, strong interest, and intense confrontation often make students’ emotions show occasional excitement, tension, laziness, and depression, which affects their next stage of learning and training, and ultimately has a certain impact on the teaching effect of comprehensive physical education courses (Viner et al., 2019).

The influence of sports on improving college students’ adverse emotions:

1. The impact of sports on the tension and excitement of college students in school.

From a physiological perspective, physical exercise can increase the secretion of adrenal glands and promote the secretion of a chemical substance similar to morphine in the brain, namely endorphin, which can create a sense of pleasure and reduce the level of negative emotions such as depression, anxiety, and tension (Bhattacharya et al., 2023). Sports activities can effectively alleviate and eliminate negative emotions such as tension, anger, fatigue, depression, and panic among college students, and improve their physical and mental health levels. In neuropsychological experiments, when people exercise, the right hemisphere responsible for emotions is immediately activated, making them energetic in a joyful atmosphere. Moderate physical exercise can help people lose weight, improve their physical fitness and image such as speed, strength, endurance, flexibility, and agility, and thereby enhance their physical self-esteem. Regular physical exercise can have a positive impact on a person’s willpower and transform it toward a positive side. When engaging in sports activities, it takes countless efforts to achieve certain goals, such as throwing the ball into the basket, kicking the ball into the net, and performing a certain action that meets the standards.

2. The inhibitory effect of entertainment in sports on negative emotions.

Sports belong to a noble form of entertainment, as their popularity can enrich people’s spiritual life, promote positive emotional development, and reduce boring pranks, gambling, fights and other low-level fun activities, preventing negative emotions caused by these stimuli (Ghalib et al., 2024). With the development of society and electronic technology, manual labor can cause mental stress. Occupational diseases in the United States, such as neuroticism, periodic migraines, and excessive mental stress, caused 390,000 people to suffer from mental stress in 1980. As a result, the UK lost 2.5 million staff in 1960, and the mental stress in country is also increasing. Brain stress has taken a heavy toll on the human body, and the means to pastime and relieve mental stress are sports and exercise, both of which can be combined to provide full relaxation.

### 2.3 Athletes’ emotions and motivational behaviors

The most commonly experienced emotion during sports activities is a state of mental excitement. Athletes feel full of strength and have a desire to express their strength. The entire physiological and psychological activity process needs to be coordinated, in order to maximize the physical fitness and technical level of athletes. The hyperactive state occurs mostly during intense moments of sports competition, where the emotional intensity is similar to that of enthusiasm, but still allows for control of one’s behavior, clear thinking, accurate judgment, and concentration. An athlete’s state of emotional intoxication is defined as being focused on the process, not feeling fatigue, not feeling injury, not feeling pain, and being able to respond in a timely manner. This emotional state is evident in ball games. There are three types of mood formation in sports competitions: one is to point directly at the opponent in singles matches; the second is internal competitions, such as running, roller skating, swimming, shooting, etc. The third is “self-competition,” which is reflected in the pursuit of “self” (Palmateer and Tamminen, 2018). Competitive state is a key factor in stimulating athletes’ potential and achieving excellent results. As an athlete, a sense of honor, pride, obligation, and responsibility is the most profound and complex high-level social emotion in sports competitions. It is the driving force to overcome difficulties, internal and external obstacles, and negative emotions.

When engaging in sports activities, corresponding measures can be taken to eliminate or alleviate negative emotions. By adjusting the speed and depth of breathing, athletes can effectively improve their emotions, because mood and breath are closely related. By maintaining a balance between oxygen and carbon concentrations in the bloodstream, the nervous system can quickly restore balance. It is necessary to gradually make one’s breathing uniform and rhythmic, in order to achieve the goal of stabilizing one’s mood. The expression regulation method refers to the conscious change of an athlete’s face, posture, and modality, in order to adjust their emotions accordingly. Due to the significant relationship between emotional state and human modality, changes in emotions can also lead to a series of physiological processes. Massage adjustment method: Massage can relieve meridians, promote blood circulation, relax muscles, and increase skin and muscle temperature. It can also adjust sensory nerves, stabilize one’s emotions, overcome excessive tension and anxiety, and restore one’s activity ability, thereby improving efficacy.

The suggestive regulation method refers to the psychological influence on participants through a “self-centered” approach. In competitive sports, psychological counseling is the most effective means of psychological regulation. It is the use of external information carriers that represent all things or phenomena within and outside the body’s environment to have an impact on human psychological processes, and the use of psychological evaluation systems to scientifically process various information from the outside world, allowing athletes to generate positive associations and a sense of empowerment, thereby enhancing their confidence in achieving excellent results and adjusting their emotional state. In experiments on sports psychology, it has been confirmed that imagery can quickly and effectively improve the directionality of attention toward actions, thereby eliminating negative psychological emotions and enhancing confidence. It can also play a role in strengthening actions and regulating emotions (Yaghoubi et al., 2019).

## 3 Introduction to athlete emotion algorithm

### 3.1 Particle swarm optimization (PSO)

PSO algorithm belongs to swarm intelligence optimization algorithm and is a new evolutionary algorithm (EA) developed in recent years (Shami et al., 2022; Gad, 2022). The swarm intelligence optimization algorithm mainly simulates the clustering behavior of insects, herds of animals, schools of birds, and schools of fish. They search for food in a collaborative manner, and each member of the group continuously adjusts their search direction based on their own experience and the experience of other members. A significant feature of swarm intelligence algorithms is the collaborative search of clusters through their swarm intelligence, thereby finding the optimal solution. The difference between the PSO method and the simulated annealing method is that the PSO method also starts from a class of random problems, and then continues to carry out iterative search. This method uses the adaptability value as the evaluation standard for the solution results, but it is much simpler than the genetic algorithm. There is no “crossover,” “mutation” and other problems. Instead, it seeks global optimization based on the current maximum adaptability value (El-Shorbagy and Hassanien, 2018).

The PSO algorithm has the advantages of fast convergence speed, fewer parameters, and easy implementation of the algorithm (for high-dimensional optimization problems, it converges to the optimal solution faster than genetic algorithm), but it also has the problem of falling into local optimal solutions.

**Figure 1:**
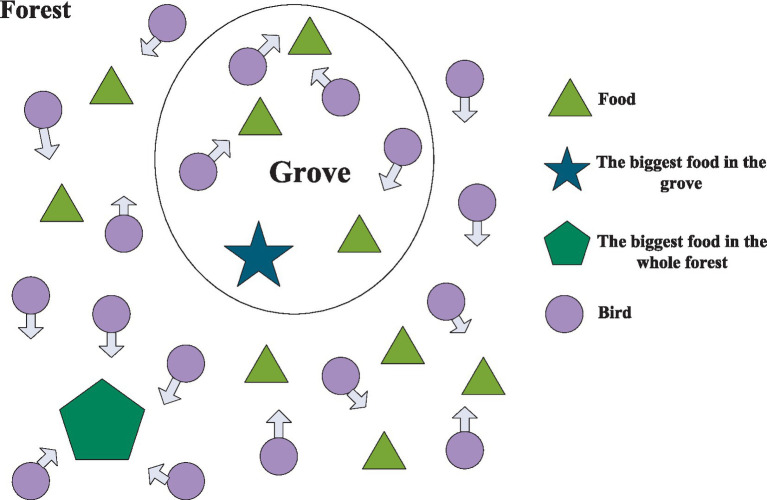
PSO algorithm diagram.

The idea of PSO algorithm originates from the study of the foraging behavior of bird populations. Among them, bird populations find the optimal destination through collective information sharing, as shown in Figure 1.

One imagines birds casually searching for food in the woods, where they hope to find the most food. However, no bird can accurately find the place with the most food, and can only feel the approximate direction of the food. Each bird searches in its own direction and records the location where it has found the most food during the search. At the same time, all birds share the location and amount of food they have found each time, so that the bird group knows which location currently has the most food. During the search process, each bird would adjust its search direction based on the location with the most food in its memory and the location with the most food recorded by the current bird group. After a period of searching, the bird flock can find which location in the forest has the most food (the global optimal solution).

The initialization process of particle swarm is a new kind of particle swarm. They describe the characteristics of particle swarm with parameters such as position, speed, fitness, etc. (Sengupta et al., 2018).

At each iteration, the particle updates its speed and position based on individual and population limits. The updated formulas are shown in Equations 1, 2.


(1) $$V_{\mathrm{id}}^{k+1}=\omega V_{\mathrm{id}}^{k+1}+c_{1}r_{1}(P_{\mathrm{id}}^{k}-X_{\mathrm{id}}^{k})+c_{2}r_{2}(P_{\mathrm{id}}^{k}-X_{\mathrm{id}}^{k})$$

(2) $$X_{\mathrm{id}}^{k+1}=X_{\mathrm{id}}^{k}+V_{\mathrm{id}}^{k+1}$$

The idea of PSO is relatively simple, mainly divided into initializing particle swarm, evaluating particles, that is, calculating fitness, finding individual extremum, finding global optimal solution, and modifying particle speed and position.

### 3.2 KNN algorithm

KNN is a statistical-based data mining algorithm that predicts unknown feature quantities of current data by searching for one or more feature quantities that are closest to the current data from a set of historical data. Therefore, it has the characteristics of being straightforward and does not require prior statistical knowledge. At the same time, KNN algorithm is suitable for two different application scenarios of classification and regression (Lubis and Lubis, 2020; Suyal and Goyal, 2022).

For the K-neighborhood algorithm, it can be understood as: assuming there is a training dataset, for a new input instance, it is necessary to find the K instances closest to the instance in the training dataset. Most of these K instances belong to a certain class, so the input instance is classified as this class. From this description, it can be seen that the KNN algorithm only stores existing training samples. When new query samples appear, a set of similar samples is extracted from memory to classify the new query samples. The classification feature of KNN algorithm is called negative learning method. Learning algorithms with the same characteristics also have local weighted regression. This method does not require one or more estimates for all samples in the sample space, but only requires local and different estimates for each sample. The corresponding classification algorithm is called active learning method, such as SVM, neural network, etc. Its characteristic is that before the arrival of a new query instance, the objective function for similar judgment is summarized through training the instance.

K-nearest neighbors can also be applied to regression tasks. When KNNs make classification predictions, they usually use a majority vote method, which means that the one with the highest number of classes among the K samples in the training set is the one with the highest number of predicted classes. When using KNNs for regression, the average method is usually used, which is to use the average output of the nearest K samples as the regression prediction value. Due to the small difference between these two methods, this article would focus on the KNN classification method and apply it to KNN regression analysis.

The advantages of KNN algorithm mainly lie in four aspects. Firstly, KNN algorithm is an online technology, where new data can be directly added to the dataset without the need for retraining. The second is that the KNN algorithm is theoretically simple and easy to implement. Thirdly, it has high accuracy and strong tolerance for outliers and noise. The fourth feature is that the KNN algorithm, like perceptron and logistic regression, is also very helpful for multiple types of classification.

In response to the shortcomings of the KNN method, when classifying a “point,” it requires a global operation, which results in a relatively large computational workload for the KNN method on a large sample set. Moreover, the KNN method is likely to cause “curse of dimensionality,” that is, when calculating distance in high-dimensional space, the calculation result of KNN method would be very far away. In the case of imbalanced samples, the prediction bias can be significant, so the size of the k value needs to be determined through experience and cross validation (Isnain et al., 2021). Cross validation or grid search can be used to select k values. When k is large, the model has a larger error and is less affected by noise, while when it is too large, it can cause the model to not conform. When the value of k is small, the deviation of the model would be large, and when the value of k is small, it may lead to overfitting of the model.

### 3.3 Basic SVM algorithm

SVM is a class of two classification models, and its basic model is a linear classifier with the largest interval in the feature space (Shang et al., 2025; Jiang and He, 2023), which is different from perceptron. It also incorporate core technologies, making them essentially a non-linear classification tool. The learning strategy of SVM is interval maximization, which can be described as a quadratic optimization problem, and is equivalent to the minimization of regularization key value loss function (Ghaddar and Naoum-Sawaya, 2018). The learning algorithm of SVM is an optimization algorithm used to solve convex quadratic programming problems.

The basic idea of SVM algorithm is to map data into a high-dimensional space, and find a hyperplane in the space, so as to maximize the distance between various data points and the hyperplane (Zhang et al., 2025; Zhou et al., 2023). Specifically, for a given training data set, SVM would determine the best decision boundary by calculating the distance between each sample point and the hyperplane. In order to avoid overfitting and improve generalization performance, SVM also introduces a kernel function, which can map linearly indivisible data to high-dimensional space, thus realizing nonlinear classification.

**Figure 2:**
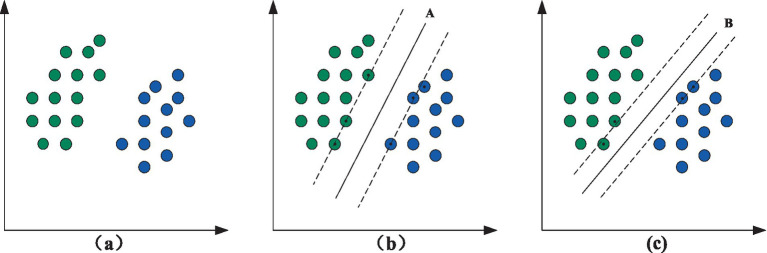
Model diagram of SVM.

The problem that SVM need to deal with is a typical second class problem. In Figure 2, it can be seen that:

In pattern recognition, the distance between two 2D data points is significant, as shown in Figure 2a. They can be divided by a line, which is the so-called linear separability problem. However, it is evident that there is more than one straight line between these two data types. Figures 2b,c show two types of classification patterns, A and B, bounded by black solid lines and referred to as “decision surfaces.” Each decision corresponds to a Linear classifier. Although the classification performance of these two methods is generally consistent based on existing data, the classification performance of these two methods may differ when considering other possible data.

The partition plane mentioned above is called “hyperplane” in SVM and can be described by the following linear formula, as shown in Equation 3:


(3) $$w^{T}x+b=0$$

Among them, $w=(w_{1},w_{2},\ldots ,w_{d})$ is the normal vector, and b is the offset term. The division of the hyperplane can be recorded as $\left(w^{T},b\right)$. At this point, the distance from x to this point can be expressed by the following formula, as shown in Equation 4:


(4) $$\gamma =\frac{|w^{T}x+b|}{\left\|w\right\|}$$

Among them,


$$\|w\|=\sqrt{w_{1}^{2}+\ldots w_{n}^{2}}.$$

The process of SVM algorithm mainly includes:

1. The preprocessing of data includes data cleaning, feature extraction, and feature extension.
2. Feature maps use a kernel function to map data onto a high-dimensional space.
3. The calculation of hyperplane is to find a hyperplane in a high-dimensional space, so as to maximize the distance from various data points to the hyperplane.
4. Prediction is the use of learned models to classify new samples.

## 4 Results and evaluation of athlete emotion recognition

### 4.1 Data selection for athlete emotion recognition

In order to make the body’s emotional analysis of athletes more accurate from the perspective of PH, the PSO, KNN algorithm and SVM algorithm introduced above were combined for feature selection. The combination of algorithms is mainly the combination of PSO and KNN algorithm, and the combination of PSO and SVM algorithm. The experimental data analysis is mainly based on the health quality and psychological status of athletes in M Club. A total of 30 athletes from the club were selected for data, including physiological indicators such as heart rate (HR), galvanic skin response (GSR), respiratory rate (RR), blood pressure (BP), blood volume pulsation (BVP), electrocardiography (ECG) physiological signals, and pupil diameter (PD). Based on the data of these 30 athletes, 191 effective samples were obtained, including 30 calm samples, 27 irritable samples, 23 excited samples, 21 bored samples, 30 low tension samples, 30 moderate tension samples, and 30 high tension samples. To eliminate physiological data differences between individual subjects, the calm samples were removed to obtain baseline physiological sample data. The final data used was summarized as 71 emotion samples and 90 tension level samples.

### 4.2 Combination evaluation of PSO and KNN algorithm

The combination of PSO algorithm and KNN algorithm is called PSO-KNN algorithm for short (Han, 2022; Yadav et al., 2024). When PSO-KNN algorithm analyzes the emotional and motivational behavior of the body to athletes from the perspective of PH, it should first analyze the emotional state of athletes, such as what kind of emotion the state would cause to athletes when the athletes appear in the body. The article was divided into a training set and a testing set based on 71 samples, mainly to identify whether the PSO-KNN algorithm can recognize the emotional state of athletes in different states when they experience physical conditions.

**Figure 3:**
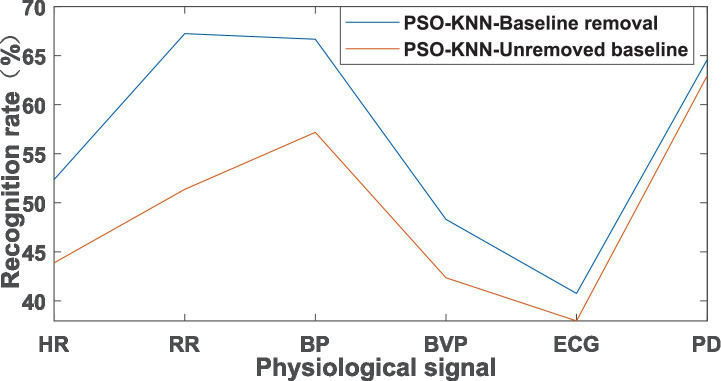
Recognition rate of PSO-KNN algorithm in emotional states.

The emotional states of the athletes were analyzed, and 71 samples were divided into 41 in the training set and 30 in the test set. The samples with the baseline removed and the samples without the baseline removed were analyzed separately, and the obtained recognition rate results are shown in Figure 3.

**Figure 4:**
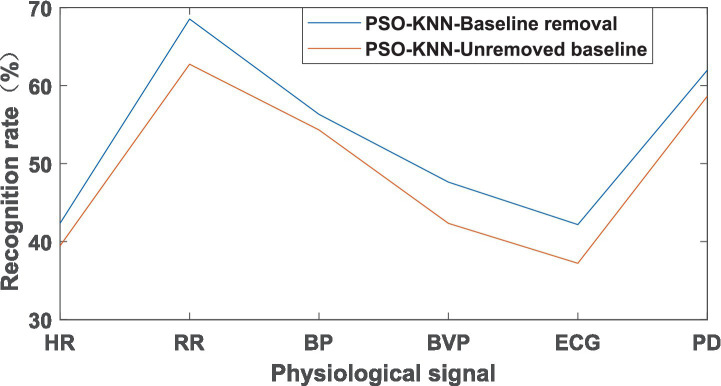
Recognition rate of PSO-KNN algorithm under stress level.

The 90 samples were divided into 63 training sets and 27 test sets. The 63 training sets were divided into 21 “low tension,” 21 “moderate tension,” and 21 “high tension” samples; the 27 test sets were divided into 9 “low tension,” 9 “moderate tension,” and 9 “high tension” samples, and the results of recognition rate were shown in Figure 4.

Through the above analysis, it can be seen that the recognition rate after removing the baseline was higher than that without removing the baseline, whether in the analysis of emotional states or in the analysis of tension levels. This indicates that removing the baseline can improve the recognition rate in emotional states and tension levels.

### 4.3 Combination evaluation of PSO and SVM algorithm

**Figure 5:**
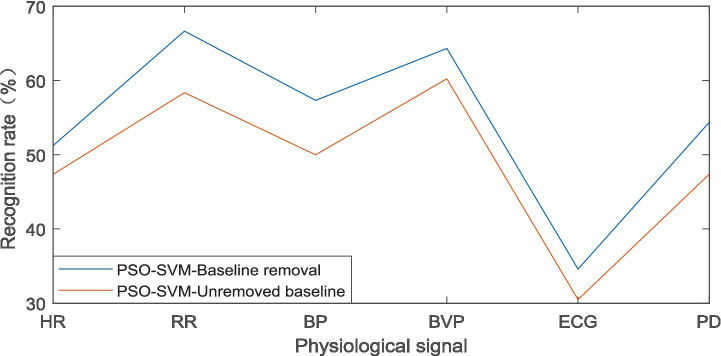
Recognition rate of PSO-SVM algorithm in emotional states.

The PSO was combined with KNN algorithm, which was called PSO-SVM algorithm for short. It also analyzed the emotional state and tension of athletes. The 71 samples under emotional state were divided into 41 training sets and 30 testing sets, and 41 training sets were further divided into 17 “irritable” samples, 13 “excited” samples, and 11 “bored” samples; the 30 test sets were further divided into 10 “irritable” samples, 10 “excited” samples, and 10 “nervous” samples. The samples with and without the baseline were analyzed separately, and the recognition rate results obtained are shown in Figure 5.

**Figure 6:**
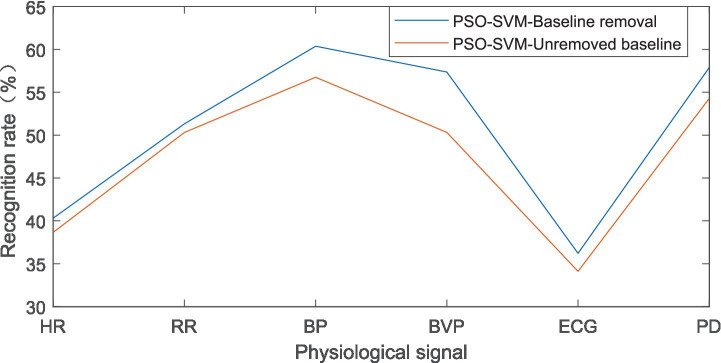
Recognition rate of PSO-SVM algorithm under stress level.

To analyze the tension level of the athletes, the 90 samples were divided into 63 training sets and 27 test sets. The 63 training sets were divided into 21 “low tension,” 21 “moderate tension” and 21 “high tension” samples; the 27 test sets were divided into 9 “low tension,” 9 “moderate tension” and 9 “high tension” samples, and the results of recognition rate were shown in Figure 6.

Based on the above results, it can be concluded that in the PSO-SVM algorithm, the recognition rate under emotional state was the highest at 66.66% when removing the baseline. Among them, the physiological signal was respiratory rate (RR); the recognition rate was the lowest at 34.58%, and the physiological signal here was the electrocardiogram. At the level of tension, the highest recognition rate was 60.37% when removing the baseline. Among them, the physiological signal was blood pressure (BP); the minimum recognition rate was 36.21%, and the physiological signal here was an electrocardiogram. It can be considered that respiratory rate (RR) and blood pressure (BP) can help identify tense emotions under stress.

### 4.4 Results of athlete emotion recognition

The stress models under emotional and tense states were analyzed using the PSO-KNN and PSO-SVM algorithms, and all results were summarized. The results are shown in Table 1.

**Table 1:** Summary of recognition rates under multiple states.

State category	Algorithm	Average recognition rate (%)
Three emotions	De-baseline (PSO-KNN algorithm)	56.66
	Unbaselined (PSO-KNN algorithm)	49.28
	De-baseline (PSO-SVM algorithm)	54.75
	Unbaselines (PSO-SVM algorithm)	48.96
Three levels of tension	De-baseline (PSO-KNN algorithm)	53.16
	Unbaselined (PSO-KNN algorithm)	49.11
	De-baseline (PSO-SVM algorithm)	50.58
	Unbaselines (PSO-SVM algorithm)	47.40

From the summary in Table 1, it can be seen that the PSO-KNN algorithm and PSO-SVM algorithm had higher recognition rates than those without removing the baseline under the condition of removing the baseline. The average recognition rates of PSO-KNN algorithm and PSO-SVM algorithm without removing the baseline under emotional states were 56.66 and 54.75%, respectively; the average recognition rates of PSO-KNN algorithm and PSO-SVM algorithm with baseline removal under tension were 53.16 and 50.58%, respectively. It can also be considered that the PSO-KNN algorithm has more advantages and higher accuracy in analyzing stress-related emotions.

## 5 Conclusion

This paper introduced the PH perspective, the impact of physical exercise on people’s emotions, and the basic contents of athletes’ emotions and motivational behaviors, and then introduced PSO algorithm, KNN algorithm, and SVM algorithm. Then the PSO was combined with KNN algorithm and SVM algorithm respectively, and the combined PSO-KNN algorithm and PSO-SVM algorithm were analyzed. Finally, it was found that the data under the baseline is more accurate, and PSO-KNN algorithm has more advantages than PSO-SVM algorithm. However, there are still shortcomings in the article, such as not having enough samples in the data sample. Due to the limited sample data, the obtained data is not very accurate. Therefore, in future research, sample data can be added.

## Data Availability

The original contributions presented in the study are included in the article/supplementary material, further inquiries can be directed to the corresponding author.
